# Community Faecal Management Strategies and Perceptions on Sludge Use in Agriculture

**DOI:** 10.3390/ijerph17114128

**Published:** 2020-06-10

**Authors:** Matthew Mamera, Johan J. van Tol, Makhosazana P. Aghoghovwia, Gabriel T. Mapetere

**Affiliations:** 1 Faculty of Natural Sciences, Department of Soil, Crop and Climate Sciences, University of the Free State, Bloemfontein 9301, South Africa; vantoljj@ufs.ac.za (J.J.v.T.); AghoghovwiaMP@ufs.ac.za (M.P.A.); 2 Faculty of Social Science and Humanities, Department of Sociology, University of Fort Hare, Eastern Cape 5700, South Africa; gmapetere@gmail.com

**Keywords:** pit latrine, faecal sludge, bacteria pollution, biochar, water quality

## Abstract

Most people in rural areas in South Africa (SA) rely on untreated drinking groundwater sources and pit latrine sanitations. A minimum basic sanitation facility should enable safe and appropriate removal of human waste, and although pit latrines provide this, they are still contamination concerns. Pit latrine sludge in SA is mostly emptied and disposed off-site as waste or buried in-situ. Despite having knowledge of potential sludge benefits, most communities in SA are reluctant to use it. This research captured social perceptions regarding latrine sludge management in Monontsha village in the Free State Province of SA through key informant interviews and questionnaires. A key informant interview and questionnaire was done in Monontsha, SA. Eighty participants, representing 5% of all households, were selected. Water samples from four boreholes and four rivers were analyzed for faecal coliforms and *E. coli* bacteria. On average, five people in a household were sharing a pit latrine. Eighty-three percent disposed filled pit latrines while 17% resorted to closing the filled latrines. Outbreaks of diarrhoea (69%) and cholera (14%) were common. Sixty percent were willing to use treated faecal sludge in agriculture. The binary logistic regression model indicated that predictor variables significantly (*p* ˂ 0.05) described water quality, faecal sludge management, sludge application in agriculture and biochar adaption. Most drinking water sources in the study had detections ˂1 CFU/100 mL. It is therefore imperative to use both qualitative surveys and analytical data. Awareness can go a long way to motivate individuals to adopt to a new change.

## 1. Introduction

Globally, governments have a critical role to ensure quality provision of water and sanitation access to their citizens [1]. Water and sanitation are basic necessities for development worldwide [2]. In developing countries, such as South Africa (SA), most people in rural areas rely on untreated drinking groundwater sources [3] and pit latrine sanitations [4,5].

South Africa has a number of outstanding sanitation needs [6]. These were categorized in terms of backlogs of service delivery, refurbishment and extension, upgrade needs, and operation and maintenance [6]. The Department of Water and Sanitation (DWS) found adverse gaps on the access to water and sanitation, mainly in the most disadvantaged communities in SA [4,6]. The disintegration and the absence of a single national body leading in the sector resulted in particular challenges in terms of the coordination and upholding of sanitation norms and standards [6]. Sanitation problems must be resolved in a manner that aids in connecting the existing gap between governance, sanitation engineers, and communities [2]. Sanitation challenges within the sub-Saharan region has led to recurring epidemics of sanitation-related diseases [7]. Use of pit latrines can be beneficial, even though there are concerns that they may cause human and ecological health impacts associated with microbiological and chemical contamination [8]. A minimum basic sanitation facility for rural areas should enable safe and appropriate treatment and/or removal of human waste in an environmentally sound manner [6]. Therefore, integrated trans-disciplinary approaches and the development of a language that both communities can understand and develop ownership for are therefore important [2]. 

Pit latrine sludge in South Africa is usually emptied and disposed off-site as waste [9,10]. The removal of sludge from pit latrines in the developing world is faced with challenges of high operation costs which causes high sanitation costs [2,11,12,13,14]. In situations where sludge disposal is costly, latrines are closed on-site [15]. Proper sludge management practices can solve some of the socio-economic challenges in communities such as disease outbreaks and water resource contamination [13]. South Africa has reported cholera outbreak cases in rural areas due to poor access to safe drinking water and sanitation systems [1]. In Free State, QwaQwa in particular, a backlog in securing access to water since 2016 led to a community protest to resolve the gap in water supply which still largely exists [16]. There is a need to create sustainable services in pit latrine maintenance and water quality [13]. However, it is important from the onset to understand the complexity of decision making within a community [17]. Socio-economic factors among other variables have an impact on development and implementation of any technology [18,19]. Sociological approaches therefore can be important to understand community norms and behaviour [17]. Despite having knowledge of potential sludge benefits, most communities in South Africa are reluctant to use it [20]. 

Productive sanitation systems that produce renewable energy from biogas or organic fertilizer from excreta and wastewater must be incorporated in the overall city planning [13]. Faecal sludge management (FSM) methods in South Africa have been adapted [13]. Such methods include those which change solid, pit latrine and other sludge into a pasteurized, dry, “handleable” product utilized for agricultural purposes [13,14]. Studies have shown that acceptance and adoption of new practices within a community depend on the additional benefits the technology provides [21,22]. Community mobilization and awareness can promote use and improvements in latrines [23]. Introduction of community-led total sanitation participation makes sustainable approaches which improve water quality, hygiene, sanitation, and sludge management attainable [24]. Proper management of sludge can lead to development and delivery of innovative concepts with value chain products and sustainable sanitation [25]. Creation of incentives and turnover can be realized through return of nutrients from human excreta as safe agricultural inputs for farmers [14,25]. Other viable methods such as biochar use in sanitation and soil improvement have potential merits [26,27,28]. Biochar is a highly adsorbing material made from any organic biomass using high temperatures by a thermal degradation process called pyrolysis [26,27,28]. Most of the research on biochar in South Africa is focused on the potential of biochar to ameliorate soil fertility for agricultural purposes [26]. There are however several potential benefits of also incorporating biochar in faecal sludge management studies [27,29]. The advantages include, removal of microbiological pathogens such as *Escherichia coli* [29]; reduction in nitrogen leaching [26]; increased pH and cation exchange capacity (CEC) [27,28]; assist in dehydrating excreta because of its high water holding capacity [26,29]; act as a barrier to prevent organic pollutants and heavy metals from percolating into groundwater aquifers [27,29]. Such benefits can only be realized by understanding the community perceptions and faecal sludge management in pit latrines.

This research aimed to capture social perceptions regarding latrine sludge management and perceived water quality, as well as perception on treated faecal sludge use in agriculture. The sociological survey is supplemented with physical measurements of organic pollution of water resources in the study area. 

## 2. Materials and Methods 

### 2.1. Location of the Study

**Figure 1:**
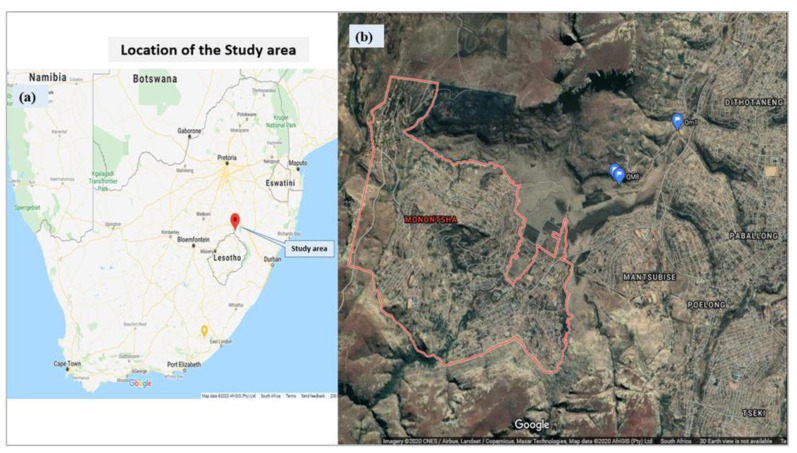
(**a**) and (**b**). Map showing the study location.

The field study and surveys were conducted in the eastern Free State, South Africa (Figure 1). The study sites are located in Monontsha, a rural village of Maluti-a-Phofung local municipality, Thabo Mofutsanyana District Municipality (−28.554257, 28.722113), which formed part of the former QwaQwa homeland. The site covers an area of 8.06 km^2^, with a population of 5552 (688.63 per km^2^) and 1552 (192.25 per km^2^) households [30]. Only households relying on Ventilated Improved Pit latrines (VIP) and Un-Improved Pit latrine (UN-IP) sanitations were included in the sociological surveys.

### 2.2. Field Study

A key informant interview and questionnaire based on guided questions was done with 10 community leaders (Chief and his council) and residents in the area. The pre-test was used to refine and enhance validity of the questionnaire. A similar approach to the Sanitation Focus Opportunity Ability and Motivation (SaniFOAM) framework [31] was used. The framework analyzes sanitation behaviors to design effective sanitation programs. In the study, 80 participants representing five percent of all households [30] were selected. The key informants provided general information on the management of waste from pit latrines. The information from the key informants was then fitted into a structured questionnaire survey which was used to probe what the communities do when the pit latrines fill up and whether they would be willing to reuse treated faecal sludge as soil conditioner for farming purposes. 

### 2.3. Water Resource Sampling

**Figure 2:**
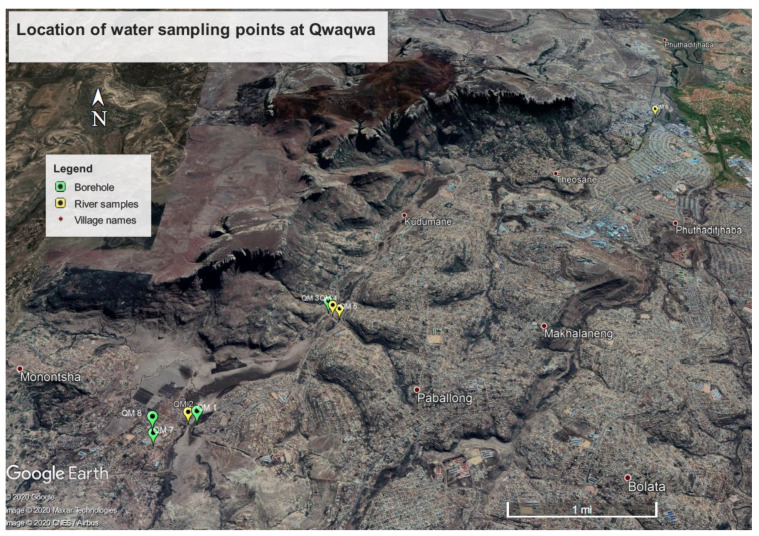
Map showing sites for the sampled water sources.

**Figure 3:**
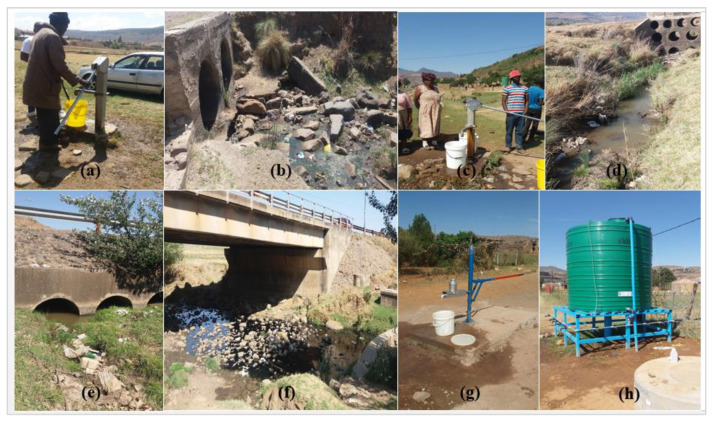
(**a,c,g,h**) Borehole drinking water sources and (**b,d–f**) river sources.

As part of the sociological survey, information regarding the occurrence, frequency, and timing of waterborne diseases (e.g., cholera and diarrhoea) was captured. The information was supplemented by collected water samples from four boreholes used for drinking water and four river sources within the study area (Figure 2 and Figure 3). 

Water samples were collected in clean plastic bottles, immediately stored at a temperature below 4 °C and analyzed within 24 h of collection for *E. coli* and faecal coliforms bacteria at the Institute for Groundwater Studies (IGS), University of the Free State. The water samples were analyzed using the membrane filtration method [32] and the polymerase chain reaction (PCR) method [33] to identify and verify the presence of bacteria. *E. coli* and faecal coliforms densities were then taken as the number of positive wells which were presented as colony forming units (CFU/100 mL). Samples were collected during three site visits to accommodate seasonal variations, i.e., before the summer rains and during the onset of the rains (October and December 2019 to February 2020). 

### 2.4. Ethical Approval 

The study was approved by the General/Human Research Ethics Committee (GHREC)-N.o-UFS-HSD2019/1012. Environmental and Biosafety Research Ethics Committee (EBREC) - N.o- UFS-ESD2019/0066.

### 2.5. Data Analysis 

The questionnaire descriptive and econometric data was analyzed with Statistical Package for the Social Sciences (SPSS, IBM Inc. version 25, Armonk, NY, USA, 2017). Prior to analysis, all the data was assigned a variable code. Variables with more than two responses were categorized to use a binary binomial logistic regression model approach. The model was used to predict the influence of socio-economic and biophysical factors on how they can affect the community water quality, sludge management, and their likelihood to use sludge in agriculture and adopt biochar uses (commercial or locally produced) in pit latrines sludge treatment. A chi-square test was used to verify the significance of the regression model and confirm if the combination of the explanatory factors explained the existing sanitation and water quality within the community. The list of dependent and predictor variable coding and description used in the binary logistic regression are shown in Table 1. In the study, simple descriptive statistics such as frequency, means, minimum and maximum values, and percentages were also calculated. Significant levels were measured at a 5% probability level. 

## 3. Results

### 3.1. Qualitative Survey 

The descriptive data showed that from the 80 participants in the study, 64% were females and 36% males. In the study, 40% of the participants were between the age of 31 and above 55 years. An average of five people in a household were sharing a pit latrine. Most people had an educational literacy, as 77.5% reached secondary level. Only 2.5% were not educated, while 5% had a higher tertiary qualification. The main source of monthly household income of the participants was government grants as 86.3% of people received an income below ZAR 4 500 (equivalent to 240 USD as per May 2020 forex exchange rate; 1 USD = 18.75 ZAR) per month. Of the total participants, only 27.6% were either self-employed or employed, while 72.4% were unemployed (Table 2 and Table 3).

Variables were further grouped into two categories; 1st group (i.e., good, dispose, willing, and adapting) and 2nd group (i.e., poor, reconstruct new latrine, not willing, and not adapting).

Overall, most people (58.8%) stated that the water source they relied on had a good quality as contrasted to 41.3% (poor quality). In the community, 83.3% disposed of their filled pit latrines sludge using the local municipality. The other 16.7% of the people resorted to closing the filled latrine and reconstructing a new latrine using a private contractor or own labor. Most participants below the age of 30 as compared to those older than 30 years were reluctant to use human treated sludge for agricultural purposes. However, overall participants in the study were willing to use treated faecal sludge (60%) in agriculture, as well as adapting biochar use (73.8%) to reduce pit latrine baseline contaminate leaching (Table 2). The main drinking water source of the participants was obtained from municipality tanks (61%) and boreholes (17.8%). Other household uses relied mainly on the river and rain harvested water. Only a few participants (4.2%) had occasional piped tap water (Table 4). Despite having as many as five water sources, respondents indicated that they could not entirely rely on the constant supply or availability of water.

Only two options were identified as a way of dealing with latrines when they filled up. In the community, the municipality (51%) was responsible for sludge disposal and the remaining 49% used private contractors. None of the respondents had the capacity to empty the latrines. Majority of the participants in the study lacked access to any sludge disposal safety equipment (98%). The main sanitation-related disease outbreak was diarrhoea (69%) and cholera (14%). The outstanding 17% of the participants had no knowledge of any disease outbreaks within the community. The study indicated that 91% of the participants planted crops while the other 9% were not involved in any agricultural practice. Of the 91% of participants, 55.5% only planted vegetables (e.g., spinach, beetroot, potatoes, cabbage, and carrots) while the additional 44.5% also had field crops (e.g., maize, beans, and pumpkins). Most of the participants (71%) had knowledge of the use of treated human sludge under agriculture. Majority of the households (83%) were willing to purchase and use biochar for sanitation and agricultural purposes. Nonetheless, only a few participants (8%) were using wood ash to reduce potential pit latrine leaching as contrasted to 92%. Most of the participants (88%) were not aware of the potential leaching from pit latrines as compared to the remaining 12%. Only 23% of the participants were using commercial detergents to treat pit latrine sludge as contrasted to 77%. Of the 23% using detergent, most people (13%) were spending less than ZAR 50 (3 USD) per month and between ZAR 51 to 100 (7%). Only 3% of the participants were willing to spend more ZAR 100 (6 USD) per month on detergent.

#### Extent of Water, Sanitation Quality, and Sludge Management in the Sampled Monontsha Village

The overall results obtained from the binary logistic regression model indicated that the tested predictor variables (socio-economic and biophysical factors) significantly described the water quality (*p* ˂ 0.01), faecal sludge management (*p* ˂ 0.04), sludge application in agriculture (*p* ˂ 0.05), and biochar adaption (*p* ˂ 0.05) in the community (Table 5). Most of the socio-economic and explanatory predictors increased the likelihood ratio of the community to adapt to new technologies (i.e., sludge and biochar) to improve their water quality, as shown with positive β values. Nonetheless, of these factors, only drinking water source and ability to purchase biochar had a significant (*p* ˂ 0.05) influence on the water quality. Faecal sludge management was highly significant at *p* ˂ 0.001 of these factors: the drinking water source and detergent price. There was a significant effect at *p* ˂ 0.05 influenced by sludge draining, access to safety equipment, disease type, crop type, and latrine sludge treatment. Application of sludge in agricultural practices was significantly (*p* ˂ 0.05) influenced by the sludge filling rate, sludge draining, type of crop, wood ash uses, and latrine detergent price. Biochar adaption was positively and significantly (*p* ˂ 0.05) affected by the human awareness on sludge use, willingness to purchase biochar, and awareness on the potential groundwater contamination from pit latrines. 

### 3.2. Bacteria Water Analysis

In general, all the river sources (Table 6) in the study had high bacteria counts (˃1 CFU/100 mL). The highest detections for both faecal coliforms and *E. coli* bacteria were seen in the river water sources above 1 CFU/100 mL. Extreme counts above 2420 CFU/100 mL in the three sampling periods were recorded in site QM 6 which is the main river draining out of the study catchment (Figure 2). In December 2019, site QM 8 was the only drinking source (Borehole) with counted above the minimum recommended threshold (˂1 CFU/100 mL), recording 3 CFU/100 mL (Faecal coliforms) and 2 CFU/100 mL (*E. coli*) (Table 6). In the successive sampling phase in February 2020, study site QM 3 (Borehole) showed evidence of faecal coliforms with a count of 1 CFU/100 mL (Table 6).

## 4. Discussion

### 4.1. Water and Sanitation Qualitative Survey 

Decision making is frequently the result of a long process in which numerous steps are essential [35]. Barnard et al. [36] and Tadesse-Yimam et al. [37], in a rural study in northern Ethiopia, suggested that there was no guarantee in the use of methods due to behavioral changes that are required which also rely on cultural norms. A study in rural Niger revealed that mostly male heads of a household make latrine operation and maintenance decisions [17]. In this study, however, in most houses (64%) women were usually responsible for household duties and latrine decisions. Maintenance of such latrines relied solely on the contribution of women. Sometimes the role of women or wives may be more informal even though they largely contribute to latrine decision making [17]. The nature of those involved directly or indirectly in the decision are related to access, use, and willingness to pay in latrine operation and maintenance [35]. Ownership of improved latrines has been strongly related to the socio-economic conditions, spatial distribution, and education status of the household head [38]. A higher education status (97.5%) of the community was shown in the health awareness to use either a VIP/UN-IP latrine. Even though other participants (2.5%) were not educated, the absence of open defecation indicated that a society can be influenced with the community norms. The Swiss TPH [38] report also confirmed that accessibility to improved latrines depends on household income and government subsidies. The survey results they obtained showed that worse-off and intermediate groups only rely on either UN-IP/VIP latrines. Similar observations were noted in this survey study. Qualitative surveys from Bangladesh, Senegal, and India showed that a higher water quality supply is directly linked to improved latrines and improved socio-economic conditions [38,39]. Similar observations were also made from this study survey data as the majority of respondents both had a considerable access to improved water and improved latrines. Swiss TPH [38] noted that in the absence of government subsidies, low income households tend to use unimproved latrines. 

Qualitative survey data in Senegal and India indicated that latrine emptying and disposal is not common in rural areas [38]. Similar findings were also seen in the qualitative survey, as some of the participants opted to reconstruct new latrines. Studies in South Africa showed that draining and disposal of each pit latrine costs between ZAR 300 (16 USD) to ZAR 1250 (67 USD) [9,10]. Most of the participants in the survey had a low monthly income which makes it unrealistic in some cases to afford the cost of draining and disposal. Nonetheless, in the context of South Africa, the qualitative survey data also showed that government support through municipality can promote pit emptying. However, Mjoli [40] and Tissington [15], also working in South Africa, argued that most municipalities lack budgets and funds for latrine emptying. In most cases, rural villages in developing countries have a low capacity to pay for latrines [41]. This might have been another reason why most of the respondents (77.5%) in the study were not eager to spend much on pit sludge treatment (Table 4). Swiss TPH [38] reported similar findings as they confirmed 18.6% as compared to a total of 19.6% relying on simple VIP latrines in their study were not able to pay anything towards latrines. In another study, Barnard et al. [36] aligned the constraints to cost of latrines (59.3%) and inadequate savings (34.1%). Moreover, they confirmed in a focus group that a weak capacity to pay exists, as 24.3% of the respondents did not consider latrine disposal and renovation a priority. Jenkins and Scott [37] studied the barriers to latrines access in Ghana and found that high costs and competing priorities were among the main constraints. In this study, commercial detergents or any other pit latrine sludge treatment material were also viewed as a competing priority with 78% of the respondents. 

An average of five people in each household filled a latrine in five years as indicated by the qualitative survey. Similar findings in SA were reported by Still and Foxon [9], Brouckaert et al [10], Department of Water Affairs and Forestry (DWAF) [42], and Seal et al [43]. In large households, members tend to be less satisfied with latrine uses [38,44]. In Tanzania, Sara and Graham [41] observed that 40% as contrasted to 50% of households with access to improved latrines barely used them. However, in this study all the respondents relied and used the available latrines. Tadesse-Yimam et al. [37] in northern Ethiopia found out that households with clean latrines were 4.3 times likely to use them. A study in East Java showed that 82.4% of households with private and clean latrines were more satisfied as contrasted to 68.3% with shared latrines [45]. This might also have been another reason people used their household latrines. Moreover, all the latrines were private to each specific household despite sharing with family members. Some of the major factors for use included dangers of feces for health (9%) and maintaining a clean environment (27.5%) [45,46]. In this survey, sanitation diseases (i.e., diarrhoea and cholera) occurred in the community according to the participants. Barnard et al. [36] also confirmed in a survey that 66% of respondents argued that there was a relationship between the utilization of latrines and better health. Findings from Ngondi [46] emphasized that there are advantages in proper latrine uses and sludge treatment practices like fly reduction (41.1%) and disease prevention (35%). Another study in the Ngohe municipality, Kenya observed that the population was keen to adapt a new behavior following awareness of the links between diarrhoea and latrines [1,47]. A cholera epidemic in the area led to an increased demand in improved latrines and better sludge disposal. Other researches in rural Tanzania [48] and in Ethiopia [49] showed that awareness in hygiene and sanitation had a nine- and two-times more likelihood, respectively, in water improvements, improved latrines, and sludge management practices. 

Sustainable methods such as biochar have been proved to be successful in sanitation and soil amendment purposes. Williams [50] had positive outcomes with an increased ammonia-nitrogen, P retention and reduction in leaching of faecal coliforms, and *E. coli* from municipal sludge treated with biochar. Studies have suggested that population growth is directly correlated to waste management [51]. In our survey, most of the participants understood the crop nutritional benefits with the combination of sludge and biochar. Awareness is important for the implementation of sustainable techniques. Studies in SA, eThekwini, Durban have seen projects involving latrine sludge treatment and by-products beneficial in cropping uses, including root crops [13]. A faecal sludge burial study in Umlazi, Durban also showed increased tree growth because of improved nutrient retention and also reduced pathogenic migrations [9]. A social survey study in Ntuzuma and Inada village Kwa-Zulu Natal (KZN) in SA to explore the perception and knowledge of farmers in the use of urine and faecal sludge showed that barriers still exist in usage. Moreover, the capacity of sludge and knowledge of the nutritional benefits is still limited. Negative perceptions due to ethical norms remain, even though farmers have the willingness to adapt [52].

### 4.2. Water Analysis 

Detection of pathogenic bacteria within drinking water sources causes a huge threat to human well-being [53,54,55,56,57]. The consequent movement of pathogens with subsurface drainage water to surface water systems has been recognized as a main pathogen transport pathway [53,56]. However, the DWAF [4] groundwater strategy report in SA highlights that groundwater is mostly safe for drinking processes without treatment. The report argues that pathogenic bacteria usually has a short survival rate in aquifers. Such findings can also explain the lower detections of bacteria in most boreholes sampled in the study. Nonetheless, results from column and field research showed that the movement of bacteria through undisturbed soils is mostly governed by macropore flow occurrences [53]. In a case where there is a shared use of VIP/UN-IP latrines as in this study, bacteria leaching can be a problem [3,55]. Physical water filtration is recognized as the principal process which restricts bacteria transport in soil [53,57]. The argument was based on the findings that bacteria range in size from 0.2–5 μm which causes soil straining and adsorption [53,58]. Looking at the results in an *E. coli* outbreak study in Canada, O`Connor [59] attributed the survival and movement of faecal bacteria to moisture and soil type, among other several factors. Such findings were similar to this study, as it shows that some of the boreholes and rivers counts increased in response to the rainfall season. Groundwater fluctuations had an effect in the population of both *E. coli* and faecal coliforms. Prevalent detections of faecal bacteria, especially in surface sources such as rivers, have been studied numerously. Niemi and Niemi [60] in Southern Finland observed counts exceeding 100 CFU/per 100 mL in surface water from non-agricultural areas and watersheds. A study in the United States in two catchments detected counts more than 200 CFU/100 mL from streams, wells, and springs [61]. Similar to this study, their results emphasized that streams exceeded the recommended threshold standards with a range between 87% and 100%. Wildi et al. [62] and Pote et al. [63] attributed the contamination of most Swiss rivers and reservoirs to mainly faecal sludge and rainwater drainage.

### 4.3. Implications

Water quality, especially drinking sources, depends on several indicators among faecal bacteria. Water use guidelines and monitoring analysis with the varying seasonal changes is critical. Characterizing a water source based on its appearance and taste only and using these as a quality indicator can be a challenge. In this study, some of the water sources had bacteria indicators above the recommended threshold (˃1 CFU/100 mL) (Table 6). According to the sensorial appearance, the respondents classified the source as a good quality due to their available resources and capacity. Verification of water quality is important and should be aligned to the socio-economic factors to improve the application of the results. In cases where an essential source which people rely on such as rivers are highly contaminated, health awareness to the users is important. This can ensure implementation of treatment improvements prior to use of that particular water source. 

Sentiments around the use of faecal sludge as manure are characterized by the lack of education around benefits. The human condition can be defined by negative perceptions of faecal waste as “useless, unnecessary and therefore undesirable” [64]. For this reason, an individual needs training to raise their awareness and thus change this perception. The obvious question mostly arises around the undesirable faecal malodor. As mentioned before, within a community one would need to be able to address the diverse demographics appropriately, the old and uneducated, the young and uneducated, as well as the educated few in a bid to alter their perceptions and encourage the use of faecal sludge. On the other hand, it would be way easier to convince any member of any community about the need to use biochar than faecal waste alone. Use of biochar and sludge together can improve water quality and sanitation, and hence can be a vital focus to motivate communities. 

In terms of creating awareness about biochar, the community members can also be made aware that the benefits of using biochar outweigh the potential risks. This is because biochar remains stable with a high adsorption potential of contaminates [27,65,66]. Reduction in sanitation diseases can become preventive rather than having to pay medical bills when there is a diarrhoea or cholera outbreak, which in most cases is recurring if the underlying causes are not resolved or addressed.

### 4.4. Policy Implications

In South Africa, policies such as the National Environmental Management Waste Act [11] and the Strategic Framework for Water Services [6] have been adapted in pit latrines. These policies state the requirements for safe sludge removal/handling in pit latrines. However, such policies are mostly focused on latrine constructions and siting with less attention on sludge management. In terms of most developing countries such as SA, it is beneficial to also include the cost of sludge management. Sludge treatment practices which increases the life span of latrines can also reduce management and disposal costs. Introduction of community programs to repurpose pit latrine sludge to acceptable commercial uses can improve people’s living standards. 

## 5. Conclusions

Water and sanitation lags still exist in South Africa among low income households similar to the study community in Monontsha village. Most people relied on municipal tanks and borehole water sources. Bridging the gap in provision of VIP latrines to the lacking population depending on UN-IP latrines can reduce sanitation-related diseases [8,48,55], i.e., diarrhoea or cholera. In the study, emptying of pit sludge through municipality services was the common management practice. Participants were keen to use biochar amendment in pit latrine sludge treatments. Use of qualitative surveys and analytical data to monitor and determine water quality for drinking water can give a preliminary view on the sources. The supplementary bacteria data indicated a potential contamination in some water sources. Most drinking water sources in the study had detections below the recommended threshold (˂ 1 CFU/100 mL). Bacteria contaminates above 1 CFU/100 mL were observed due to groundwater fluctuations. Extreme *E. coli* and faecal coliforms counts (˃100 CFU/100 mL) were observed in all river sources. Full implementation of a new technology still has setbacks because of dynamics in cultural norms within this society. Awareness can go a long way to motivate individuals to adopt to a new change. In the case of biochar use in water and sanitation and sludge treatment for agricultural purposes, most people were willing to use it after discussions of the potential benefits. Most respondents had knowledge on the use of treated faecal sludge but none of them actually used it before in any agricultural purpose. Future research should focus on application of the sludge perceptive to adapt sustainable uses and also capture more social norms on a larger scale. 

## Figures and Tables

**Table 1:** Description and unit of variables used in the binomial logistic model.

Dependent Variables	Variable Description	Expected Effect
Y *	Quality of drinking water (0 = Poor 1 = Good)	Determined by explanatory variables
Y **	Community faecal sludge management in VIP/UN-IP Latrines (0 = Empty 1 = Construct new pit)	
Y ***	Sewage sludge application in agricultural production (0 = Willing 1 = Not willing)	
Y ****	Biochar adaption in sludge treatment (0 = Yes 1 = No)	
**Explanatory variables**		
**Socio-economic characteristics**		
Gender (X1)	Gender of the participant (0 = Male 1 = Female)	
Age (X2)	Age of participant (Years)	
Household size (X3)	Number of household occupancy	
Education (X4)	Participant education level (0 = None 1 = Primary 2 = Secondary 3 = Tertiary)	
Employment (X5)	Participant employment status (0 = Employed 1 = Self-employed 2= Unemployed)	
Income (X6)	Average monthly household income (Measured in SA Rand, ZAR)	
**Social amenities: Water and Sanitation**		
Drinking water source (X7)	Source used (0 = Selected 1 = Not selected)	
Household water source (X8)	Source used (0 = Selected 1 = Not selected)	
Regularity (X9)	Water supply/ flow (0 = Regular 1 = Not regular 2 = Unreliable)	
Sanitation type (X10)	Household sanitation type (0 = VIP 1 = UN-IP latrine)	
Latrine users (X11)	Number of household members using pit latrine (Head count)	
Sludge filling rate (X12)	Period a pit latrine is used by a household (Measured in years)	
Sludge draining (X13)	Pit latrine sludge disposal (0 = Participant 1 = Community 2 = Municipality 3 = Private contractor)	
Equipment (X14)	Access to disposal equipment (0 = Yes 1 = No)	
Diseases (X15)	Perceived outbreaks in the community (0 = Cholera 1 = Dysentery 2 = Diarrhea 3 = No outbreaks)	
**Community perceptions on treated faecal sludge in crop production**		
Crops (X16)	Major crops grown (0 = Selected 1 = Not selected)	
Fertilizer (X17)	Use of fertilizer in cropping practices (0 = Yes 1 = No)	
Manure (X18)	Use of animal manure (0 = Yes 1 = No)	
Yield (X19)	Estimate crop yield (0 = Low 1 = Medium 2 = High)	
Human manure (X20)	Awareness of the use of faecal sludge (0 = Yes 1 = No)	
**Biochar amendments**		
Biochar use in latrines (X21)	Willingness to use in pit latrine sludge treatments (0 = Yes 1 = No)	
Purchasing (X22)	Willingness to buy Biochar (0 = Yes 1 = No)	
Wood ash (X23)	Use of ash in pit latrines (0 = Yes 1 = No)	
Groundwater contamination (X24)	Awareness on pit latrine water pollution (0 = Yes 1 = No)	
Pit latrine sludge treatment (X25)	Material added in latrine to reduce groundwater contamination (0 = Nothing 1 = Detergent)	
Detergent price (X26)	Cost of material applied in pit latrines per month (Measure in SA ZAR)	
β1…βn	Coefficients of independent variables X1…Xn	
α	Intercept	

**Table 2:** Socio-economic predictor variables characteristic in the study.

Variables	Response	(%)	Total (%)
Water quality	Good	58.7	100
	Poor	41.3	
Sludge management	Dispose	83.4	100
	Reconstruct latrine	16.6	
Sludge use in agriculture	Willing	60	100
	Not willing	40	
Biochar adaption	Yes	73.8	100
	No	26.2	

**Table 3:** Socio-economic characteristics of the households in the study.

Variables		Total (%)	1st Group	2nd Group
Gender (%)	Male	36.3	69.8	30.2
	Female	63.8	68.6	31.4
Age (Mean-49, Minimum-18, Maximum-80)	Below 30	21.3	69.12	30.89
	31–54	40	72.66	27.35
	Above 55	38.8	65.32	34.68
Average household sizes		4.6	5	4.7
Participant highest education level (%)	None	2.5	87.5	12.5
	Primary	15	66.67	33.33
	Secondary	77.5	69.76	30.24
	Tertiary	5	56.25	43.75
Participant employment status	Employed	11.3	52.78	47.22
	Self-employed	16.3	67.31	32.69
	Unemployed	72.5	55.33	44.67
Estimated monthly household income (%)	Below ZAR 1200	41.3	68.94	31.06
	ZAR 1200–4500	45	67.80	32.20
	Above 4500	13.8	77.27	22.73

**Table 4:** Frequencies of the significant variables on the water and sanitation in Monontsha village.

Variable		Frequencies (%)
**Water sources**	Tap water	4.2
	Rainwater harvesting	8.5
	Municipal tank	61
	River	8.5
	Borehole	17.8
**Number of Latrine users**	2–3	33.3
	4–6	58.8
	7–14	8.2
**Sludge emptying**	Municipality	51.25
	Private contractor	48.75
**Sludge disposal equipment**	Yes	2
	No	98
**Perceived disease outbreaks**	Cholera	14
	Diarrhea	69
	No outbreak	17
**Crop type**	Vegetables	55.5
	Field crops	44.5
**Awareness of human manure uses**	Yes	71.3
	No	28.8
**Willingness to purchase biochar**	Yes	82.5
	No	17.5
**Wood ash use in latrines**	Yes	7.5
	No	92.5
**Awareness for potential pit latrine pollution**	Yes	12.5
	No	87.5
**Pit sludge treatment**	Not treating	77.5
	Detergents	22.5
**Commercial detergent price**	˂ ZAR 50	12.5
	ZAR 51–100	7.5
	˃ ZAR 100	2.5

**Table 5:** Binary logistic regression of factors influencing water quality, faecal sludge management, sludge application in agriculture and adoption of biochar within a community.

Variables		Water Quality			Faecal Sludge Management			Sludge Application in Agriculture			Biochar Adoption		
		β	*p* Value	Exp β	β	*p* Value	Exp β	β	*p* Value	Exp β	β	*p* Value	Exp β
**Socio-economic**	Gender	1.45	0.19	4.25	−0.07	0.94	0.92	0.10	0.84	1.11	−0.67	0.51	0.51
	Age	0.45	0.77	1.57	−0.65	0.62	0.52	0.05	0.89	1.05	1.07	0.50	2.92
	Household size	2.00	0.20	7.08	−4.76	0.04	0.00	−0.16	0.46	0.86	0.56	0.78	1.76
	Education	8.25	1.00	1.21	−1.27	1	0.28	0.28	059	1.32	1.17	1.00	3.21
	Employment	0.14	0.54	1.06	−0.04	0.98	0.96	0.19	0.63	1.21	0.47	0.74	1.59
	Income	0.30	0.37	1.26	8.06	0.99	33	0.23	0.51	1.26	−0.84	0.56	0.43
	Drinking water												
	^1^ Tap water	-	0.97	0.00	-	0.92	17.5	-	0.99	0.00	-	0.99	1.39
	Rain harvesting	−9.89	0.99	0.00	18.4	0.99	19.7	−0.05	0.81	0.63	−19.3	0.99	0.00
	Municipal tank	−0.92	0.50	0.40	0.04	0.97	1.04	0.50	0.54	1.64	−0.54	0.67	0.58
	River	−2.61	0.14	0.07	−2.54	0.26	0.08	0.20	0.79	1.23	−0.46	0.73	0.64
	Borehole	−3.73	0.04 *	0.24	0.04	0.001 ***	1.04	0.49	0.50	1.64	−0.53	0.67	0.58
	Household water	−0.92	0.54	0.40	−0.06	0.97	0.94	0.27	0.75	1.31	−0.19	0.89	0.83
	Regularity	−0.10	1.00	0.00	−15.3	0.99	0.00	0.50	0.77	1.64	17.4	0.99	60
	Constant	0.35	0.12	1.42	−1.64	0.00	0.19	41.6	0.99	113	2.2	0.00	9
	Nagelkerke R^2^		0.66			0.61			0.24			0.33	
	Chi-squared		53.8			35.3			15.4			13.6	
	*p* Value		0.01 *			0.006 *			0.57			0.69	
**Sanitation**	Sanitation type	9.3	1.00	0.18	0.52	0.79	1.68	1.39	0.18	4.00	19.7	0.99	51
	Latrine users	0.17	0.80	1.17	−1.44	0.02 *	023	−22.6	0.99	0.00	2.32	0.21	10.2
	Sludge filling rate	0.68	0.12	0.26	0.68	0.65	1.96	1.89	0.03 *	6.59	19.6	0.99	54
	Sludge emptying	0.09	0.89	1.10	−1.68	0.03 *	0.19	−1.66	0.01 *	0.19	0.02	0.98	1.02
	Equipment	−4.31	0.99	0.00	1.94	0.02 *	27.2	20.1	1	51	0.98	1.00	2.66
	Diseases												
	^1^ Cholera	-	0.99	0.00	-	0.12	0.12	-	1	0.75	-	0.99	45
	Diarrhea	0.43	0.59	1.54	−1.50	0.01 *	0.22	−1.17	0.14	0.31	−0.83	1.00	0.44
	No outbreak	−20.8	0.98	0.00	−20.6	0.99	0.00	−1.28	0.26	0.28	−1.02	0.52	0.36
	Constant	1.07	1	2.91	−40.2	1	0.00	24.5	1	42	19.7	1	34
	Nagelkerke R^2^		0.53			0.69			0.33			0.33	
	Chi-squared		39.8			42			22.1			13.6	
	*p* Value		0.004 *			0.04 *			0.05 *			0.4	
**Agriculture**	Crops	3.64	0.04 *	2.37	1.34	0.05 *	3.82	−1.60	0.03 *	0.20	1.61	0.26	5.00
	Fertilizer	1.71	0.27	5.54	−3.93	0.18	0.02	−0.31	0.85	0.73	0.92	1.00	2.52
	Manure	0.30	0.63	1.35	−0.71	0.54	0.49	−1.26	0.08	0.28	−1.61	0.34	0.20
	Yield	−1.83	0.27	0.16	0.85	0.69	0.43	−0.30	0.83	0.74	−19.4	1.00	0.00
	Human manure use	0.29	0.63	1.35	0.72	0.59	0.49	−0.53	0.46	0.59	−2.02	0.05 *	1.82
	Constant	0.41	0.99	1.04	−13.2	1	0.00	2.22	1	9.18	79.2	0.99	241
	Nagelkerke R^2^		0.28			0.56			0.45			0.63	
	Chi-squared		18.8			32.1			31			28.4	
	*p* Value		0.22			0.07			0.05*			0.05 *	
**Pollution management**	Biochar use in latrines	−0.43	0.85	0.65	−0.24	0.82	0.79	−1.65	0.08	0.19	−36.7	0.99	0.00
	Purchasing	−1.31	0.05 *	0.27	0.37	0.77	1.44	1.07	0.30	2.90	3.6	0.01 *	95
	Wood ash	−0.74	0.68	0.48	−0.33	0.81	0.72	2.26	0.05 *	9.57	−1.60	0.23	0.20
	Water pollution	−0.38	0.93	0.68	−0.97	0.44	0.38	0.78	0.35	2.18	−1.37	0.02 *	0.26
	Pit sludge treatment	−4.51	0.82	0.00	−2.21	0.03 *	0.11	21.7	0.99	258	−19.2	0.99	0.00
	Detergent price												
	^1^ ˂ ZAR 50	-	0.44	8.89	-	0.001 ***	0.09	-	0.04 *	0.88	-	1.00	0.07
	ZAR 51–100	8.89	0.99	13.7	−1.85	0.21	0.92	23.8	0.99	21.7	−2.98	0.98	0.03
	˃ ZAR 100	9.91	0.99	15.1	0.42	0.83	1.52	−0.13	0.96	0.88	−22.5	0.99	0.00
	Constant	2.69	0.1	14.7	0.04	0.99	1.04	−22.1	0.99	0.00	22.7	0.99	70
	Observations		80			80			80			80	
	Nagelkerke R^2^		0.19			0.23			0.34			0.51	
	Chi-squared		11.8			11.6			23.4			22.3	
	*p* Value		0.16			0.17			0.003 *			0.002 *

*β* is the model intercept coefficient which is the expected mean value of Y when all predictor variables Xn = 0; Exp (β) is odds ratio which represents the constant effect of a predictor X, on the likelihood that one outcome will occur; * and *** Significance at 0.05 and 0.001 probability level; ^1^ X is the baseline variable for categorical variables in the models.

**Table 6:** Water analysis for faecal coliforms and *E. coli* bacteria contaminates (CFU/100 mL).

Site	Water Source	*Faecal coliforms*	*E. coli*	*Faecal coliforms*	*E. coli*	*Faecal coliforms*	*E. coli*
		Oct 2019		Dec 2019		Feb 2020	
*QM 1*	Borehole	˂1	˂1	˂1	˂1	-	-
*QM 2*	River	687 *	687 *	99 *	70 *	1414 *	980 *
*QM 3*	Borehole	˂1	˂1	˂1	˂1	1	˂1
*QM 4*	River	11 *	11 *	˃2420 *	˃2420 *	˃2420 *	˃2420 *
*QM 5*	River	1986 *	50 *	261 *	86 *	1986 *	1553 *
*QM 6*	River	˃2420 *	˃2420 *	˃2420 *	˃2420 *	˃2420 *	1553 *
*QM 7*	Borehole	˂1	˂1	˂1	˂1	˂1	˂1
*QM 8*	Borehole	˂1	˂1	3 *	2 *	˂1	˂1

*-Levels which exceed drinking water national standards (˃1 CFU/100 mL) according to SANS, [34].
